# Notes and News

**Published:** 1899-03-11

**Authors:** 


### Notes and News.

(Collected and Communicated.)

### HOSPITAL MEETINGS.

Royal Hospital for Diseases of the Chest, City Road.—Annual Meeting at three p.m., on Wednesday, March 15th.

Metropolitan Hospital, Kingsland Road, N.E.—Annual Meeting at two p.m., on Monday, March 20th next.

St. Mary's Hospital, W.—Annual Meeting at half-past four p.m., on Friday, March 17th.

The Dental Hospital of London, 40, Leicester Square, W.C — Annual General Meeting at half-past five p.m., on Friday, March 17th.

West London Hospital.—Annual Meeting at four p.m., on Wednesday, March 15th.

East End Mothers' Home, 390, Commercial Road.—Annual Meeting at three p.m., on Thursday, March 23rd, at St. Martin's Church Vestry Hall, Charing Cross, W.C. The Rev. Prebendary Kitto in the chair.

North London Hospital for Consumption.—Annual Meeting at four p.m., on Wednesday, March 29th, at 41, Fitzroy Square.

Lord Lister has been appointed a correspondent of the Paris Academy of Medicine.

Princess Henry of Battenberg has consented to open the new hospital buildings at Beckenham on May 16th. They have been erected in commemoration of the Diamond Jubilee.

Seldom has greater or more widespread interest centred round a sick bed than during last week, when two nations awaited day by day with deep anxiety the issue of Mr. Kipling's serious illness. Universal sympathy is felt with Mr. and Mrs. Kipling in the death of their eldest little daughter.

The following neat little joke we take from the *Practitioner*: "The Prince of Wales was not only present at the Hunterian Oration, but further honoured the college and its President by dining with them in the evening. The dinner being a private affair, no report of it was allowed to appear in the papers. But a little joke which was made on the occasion has been flying about *per ora virom*, and I think it worthy of being recorded. The company was, as usual, received by the President in a large room of the museum, a striking feature of which is the vast array of skulls which grin in countless rows upon the spectator. Glancing at these relics, a distinguished guest slily asked the President, 'Do you always receive your guests here in the *scullery*?'"

Mr. Bryant has been re-elected president of the Royal Medical and Chirurgical Society.

The foundation-stone of the Yorkshire Foresters Orphanage and Convalescent Home was laid at Bridlington on February 23rd in the presence of a large gathering.

The Duke of York has consented to preside at a festival dinner to be held at the Hotel Cecil on Wednesday, May 17th, in aid of the funds of the Royal Alfred Aged Merchant Seamen's Institution.

At the last meeting of the Medical Society of London, the President announced that the Fothergill Prize had been awarded to Dr. S. Monckton Copeman for his recent researches in reference to glycerinated vaccine.

Mr. Frederick Treves, consulting surgeon to the London Hospital, has been appointed Emeritus Professor of Surgery to the Hospital, and during the winter session will deliver a course of lectures on Clinical Surgery.

The new cottage hospital at Ashbourne, a memorial of the Diamond Jubilee, was formally opened by Lady Florence Duncombe, of Calwich Abbey, a week or two ago. The hospital has been built at the expense of Mr. T. O. Farmer.

Sir Henry Harben has promised a donation of £3,000 towards the Hampstead Hospital Building Fund on condition that £14,000 are raised by June 1st. A garden party in aid of the hospital is to be held on that date, to which Princess Christian has given her patronage. Promised donations already amount to £3,050.

The committee of the Manchester Hospital for Consumption and Diseases of the Throat have received an offer from a generous friend to build a new hospital for a hundred beds, provided that Manchester undertakes its maintenance. This means, in the opinion of the committee, a yearly sum of £3,000 exclusive of patients' payments, and this, they hope, there will be no difficulty in raising.

The Metropolitan Asylums Board has adopted the report drawn up by a sub-committee appointed to consider the question of imbecile classification and accommodation. The report recommends that in future imbecile children shall be divided into two groups, separately housed under different medical superintendents, special attention being given to the training of "improvable children," who, by the proposed arrangement, will be no longer classified with hopeless idiots. This is certainly a step in the right direction.

Philadelphia has lately been suffering from a severe outbreak of typhoid fever. Four hundred and twenty-seven cases were reported for the week ending January 28th, as compared with nine in Boston, and sixteen in New York. According to the New York *Medical Record* the outbreak is thought to be due to contamination of a reservoir with the overflow from an intercepting sewer. Of seventy-two cases which were reported on February 6th and 7th nearly two-thirds occurred in wards supplied with water from the infected reservoir.

